# The potential role of exosomal miRNAs and membrane proteins in acute HIV-infected people

**DOI:** 10.3389/fimmu.2022.939504

**Published:** 2022-08-12

**Authors:** Xin Li, Wenjing Wang, Jing Chen, Bangxiang Xie, Shumin Luo, Dexi Chen, Chao Cai, Chuanyun Li, Weihua Li

**Affiliations:** ^1^ Institute of Infectious Diseases of Integrated Traditional Chinese and Western Medicine, Beijing Youan Hospital, Capital Medical University, Beijing, China; ^2^ General Surgery Center, Beijing Youan Hospital, Capital Medical University, Beijing, China; ^3^ Beijing Institute of Hepatology, Beijing Youan Hospital, Capital Medical University, Beijing, China

**Keywords:** HIV, exosomal miRNAs, exosomal member proteins, rapid progressors, typical progressors

## Abstract

Exosomes play an important role during human immunodeficiency virus (HIV) acute infection. Yet, information regarding its cargo and its association with HIV rapid progressors (RPs) and typical progressors (TPs) remain largely unknown. In this study, exosomal miRNAs sequencing and mass cytometry were used to identify differential exosomal miRNAs and membrane proteins that participate in the pathogenesis of TPs and RPs. We discovered that miR-144-5p, miR-1180-3p, miR-451a, miR-362-5p, and miR-625-5p are associated with the TPs and miR-362-5p with the RPs. Decreased autophagy, amino acid metabolism, immune response, and IL-6 are closely related to RPs. In addition, SP1 was selected as the most significant transcription factor (TF) associated with disease progression. CD49D, CD5, CCR5, CD40, CD14, and CD86 were selected as the differential exosomal membrane proteins between TPs and RPs. This study provides valuable information for clarifying the mechanism in people with acute HIV infection.

## Introduction

Acquired immunodeficiency syndrome (AIDS) is a chronic, life-threatening condition and the most advanced stage of immunodeficiency virus (HIV) infection (1). The most common transmission routes include anal or vaginal sex, sharing needles, syringes, or other drug-injection equipment, and mother-to-child transmission (2, 3). Human immunodeficiency virus types 1 and 2 (HIV-1 and HIV-2) that cause AIDS (3) have many similarities, including intracellular replication mechanisms and transmission modes. However, HIV-1 has higher transmissibility, and AIDS caused by HIV-1 is a more aggressive type of disease (4). Moreover, HIV-1 is widely spread worldwide, while HIV-2 is mainly prevalent in countries in West Asia (5).

Antiretroviral therapy (ART) is the best treatment for AIDS, yet, ART is a lifelong therapy that can only control the activity of HIV and cannot prevent its transmission. Furthermore, not all patients respond well to HIV vaccination (6–10). Therefore, further exploring the mechanism of HIV-1 might help develop new treatments.

HIV infection can be divided into three types: typical progression (TPs), rapid progression (RPs), and long-term non progression (LTNP). The RPs is defined as an infection that appears within 3 years; TPs as an infection that appears between 8 years to 10 years after the first infection; LTNP is defined based on the number of CD4 cells > 400 after infection over 10 years (11). Studies on HIV have been mainly focused on LTNP, while TPs and the RPs have been less reported.

Exosomes are small membrane-bound nano-sized vesicles 30-150 nm in diameter that contain many biologically active molecules involved in the communication between cells and the surrounding microenvironment (12–14). MicroRNAs (miRNAs) are 21-22nt small non-coding RNA that regulate gene expression and are involved in cell proliferation, cell differentiation, cell migration, disease initiation, and disease progression (15–19). Studies have suggested that miRNA protects exosomes from degradation and guarantees their stability (20). Also, miRNAs in exosomes are considered a third way of intercellular communication that has the same importance as the cell-to-cell contact-dependent signaling and signaling *via* the transfer of soluble molecules (21, 22). Detection of exosomal member proteins can help researchers to figure out which kinds of cells secreted these miRNAs.

Prior studies suggest exosomes have an important role in HIV pathogenesis. Yet, information regarding its cargo and its association with HIV progression is not fully understood. In this study, we explored the role of exosomal miRNAs and member proteins in the pathogenesis of the TPs and the RPs in people infected with HIV-1. We also highlighted the need for new technologies that can associate a specific marker with an exosome subtype and the exosome subtype to a particular function and/or group of functions (23).

## Materials and methods

### Patients and samples

A total of 15 participants, including 5 Healthy control (HC), 5 HIV patients with TPs, and 5 HIV cases with RPs from Beijing Youan Hospital, were included between 2006 and 2016. Plasma samples were collected at 1, 6, and 12 months after HIV infection. HIV was diagnosed by the Chinese Center for Disease Control and Prevention or the department of infectious diseases in Beijing You’an Hospital. The classification of HIV was based on stages of disease progression (DP): the RPs was defined as an infection that appears within 3 years; TPs as an infection that appears between 8 years to 10 years after the first infection (24).

Peripheral blood samples from patients were collected in EDTA tubes following a regular venipuncture procedure. After centrifugation at 3,000 ×g for 15 min at 4°C, the plasma was aspirated and stored at −80°C.

### Plasma exosomes isolation (ultracentrifugation and molecular size exclusion)

The UC (ultracentrifugation) method was optimized according to the previously described approach (25). After thawing at 37°C, plasma samples were centrifugated at 3,000 ×g for 15 min to remove cell debris. Then, the supernatant was diluted by a seven-fold volume of phosphate-buffered saline (PBS), centrifuged at 13,000 ×g for 30 min, and processed through a 0.22 μm filter to remove large particles. The supernatant was then ultracentrifuged using a P50A72-986 rotor (CP100NX; Hitachi, Brea, CA, USA) at 100,000 ×g, 4°C, for 2 h to pellet the exosomes. Then, the pellet was re-suspended in PBS and centrifuged at 100,000 ×g 4°C for 2 h. After PBS washing, the exosomes pellet was re-suspended in 100 µl PBS.

The size exclusion method (SEC) was used to separate the exosomes. Briefly, after 0.8 μm filtration, 1 ml plasma sample was diluted with 1.5 times PBS and then purified by Exosupur exclusion column (Echobiotech, China) following the manufacturer’s instructions. The sample was then eluted with 0.1M PBS, and 2ml of the target fraction was collected. Finally, the exosome solution was concentrated (200 μL) through an Amicon^®^ ultrafiltration tube with a molecular weight cutoff of 100kDa (Merck, Germany).

### Characterization of exosomes

#### Nanoparticle tracking analysis (NTA)

Vesicle suspensions with concentrations between 1x10^7^/ml and 1x10^9^/ml were examined using the ZetaView PMX 110 (Particle Metrix, Meerbusch, Germany) equipped with a 405 nm laser to determine the size and quantity of particles. A video of 60-sec duration was taken with a frame rate of 30 frames/sec, and particle movement was analyzed using NTA software (ZetaView 8.02.28).

#### Transmission electron microscope (TEM) inspection

A 10 µl exosome solution was placed on a copper mesh and incubated at room temperature for 10 min. After washing with sterile distilled water, the exosome was mixed with uranyl acetate solution for 1 min. The sample was then dried for 2 min under incandescent light. The copper mesh was observed and photographed under a transmission electron microscope (H-7650, Hitachi Ltd., Tokyo, Japan).

#### Confocal laser scanning microscopy (CLSM)

The exosome supernatant was denatured in 5× sodium dodecyl sulfonate (SDS) buffer and subjected to Western blot analysis (10% SDS-polyacrylamide gel electrophoresis; 50 µg protein/lane) using rabbit polyclonal antibody CD63 (sc-5275, Santa Cruz, CA, USA). The proteins were visualized on the Tanon4600 Automatic chemiluminescence image analysis system (Tanon, Shanghai, China).

### Western blot (WB)

Exosomes were denatured in 5× sodium dodecyl sulfonate (SDS) buffer, and then rabbit anti-CD63 (sc-5275, Santa Cruz, CA, USA), CD9 (60232-I-Ig, Proteintech, Rosemont, IL), HSP90 (60318-I-Ig, Proteintech, Rosemont, IL), Alix (sc-53540, Santa Cruz, CA, USA), TSG101 (sc-13611, Santa Cruz, CA, USA) and calnexin (10427-2-AP, Promega, Madison, Wis Visualize protein detection results).

### ExoRNA isolation and RNA analysis

The total RNA of plasma exosomes was separated using the miRNeasy^®^ Mini kit (Qiagen, cat. No. 217004) following the manufacturer’s instructions. The concentration and purity of RNA were evaluated by the RNA Nano 6000 Assay Kit of Agilent Bioanalyzer 2100 System (Agilent Technologies, CA, USA).

### Library construction and sequencing

One to 500 ng of total RNA was used for small RNA libraries preparation by QIAseq miRNA Library Kit (Qiagen, Frederick, MD), which assigned a unique molecular index (UMI) sequence to each cDNA *via* reverse transcription. The sample index sequence was added during library amplification. The library quality was evaluated by Agilent Bioanalyzer 2100. Index-labeled samples were clustered in the acBot Cluster Generation System using TruSeq PE Cluster Kitv3-cBot-HS (Illumina, San Diego, CA, USA), and then paired-end sequencing was performed on the Illumina Hiseq platform.

### miRNA analysis

#### miRNA identification and differential expression analysis

Bowtie was used to compare clean reads with Silva, GtRNAdb, Rfam, and Repbase databases to filter out rRNA, tRNA, snRNA, snoRNA, other ncRNAs, and repetitive sequences, respectively. The remaining reads were used for comparisons with miRbase and the human genome (GRCh38) and the identification of known miRNAs and prediction of new miRNAs. The number of reads for each miRNA was corrected by UMI and then normalized by the TPM method.

### Target gene prediction

Target gene prediction method and software: the intersection of the prediction results of RNAhybrid and miRanda. Threshold parameter: RNAhybrid minimum free energy threshold: -30, miRanda scoring threshold: 150, miRanda minimum free energy threshold: -30.

### Enrichment analysis of GO and KEGG pathways

Gene functions and signaling pathways were annotated using GO full name (GO) and KEGG full name (KEGG) databases. The clusterProfiler package was used for GO and KEGG enrichment analysis of differential genes. The significant p-value was obtained by the algorithm of hypergeometric distribution, and the q-value was obtained by BH correction.

### Weighted co-expression network construction and identification of key modules

Exosomes miRNA co-expression networks were built according to the procedure of the WGCNA package in the R language (26). After filtering expression data using TPM>1 & cv > 0.5, 693 miRNAs were obtained. Primarily, we constructed the gene expression similarity matrix based on calculating the absolute value of Pearson’s correlation coefficient between gene pairs. Next, the similarity matrix was transformed into an adjacency matrix using a power adjacency function, which encodes the strength of the connection between node pairs (27). The power β = 4 was chosen based on the scale-free topology criterion when the R^2^ value was approximated to 0.81. A topological overlap matrix (TOM), which was based on the adjacency matrix, was used to identify miRNA modules, while a dynamic tree-cut algorithm was used to allocate miRNAs to different modules. The minimal module size was set at 30, and a threshold of 0.1 was selected to merge similar miRNA modules.

### Sample preparation of mass cytometry

Plasma exosome samples were collected from HIV-infected people and healthy donors. All samples were cultured with metal-conjugated antibodies in 50 μL CSB for 30 min at RT. After triple washing in CSB, exosomes were incubated with 0.05 μm intercalator in fix and perm buffer (Fluidigm) at 4 °C overnight.

### Data acquisition in Helios

After being cultured with an intercalator, exosomes were washed three times with deionized water. Before the acquisition, samples were resuspended in deionized water containing 10% EQ 4 Element Beads (Fluidigm). Data acquisition was performed on a Helios mass cytometer (Fluidigm). The original.FCS data were normalized and.fcs files were collected for each individual. All.fcs files were uploaded into Cytobank, and data cleaning was performed according to previous research. Also, tSNE analyses were performed as previously described.

### Statistical analysis

The data were analyzed by using R software (version 3.6.1, R Project for Statistical 155 Computing) and GraphPad Prism 7 (GraphPad Software, San Diego, California). For the two independent samples, Student’s t-test (normally distributed data) and the Mann–Whitney U test (non-normally distributed data) were used to identify the significant differences. The mean intensity of protein expression was calculated by Cytof software. The Kruskal-Wallis H test was used for multiple independent samples. P-value <0.05 and Q value <0.05 were considered to be statistically significant.

## Results

### The baseline clinical characteristics of participants and identification of exosomes

**Figure 1:**
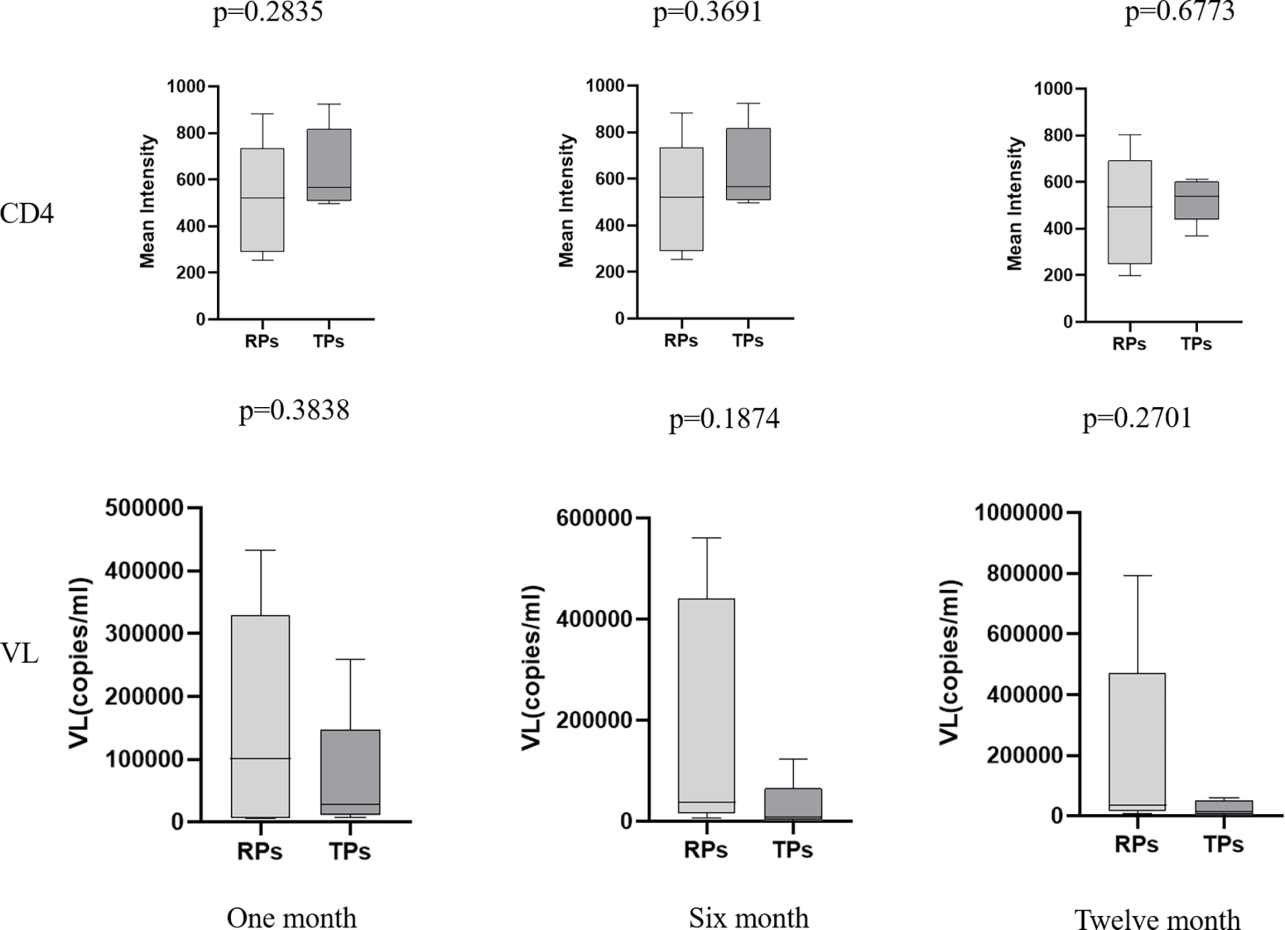
The comparison of CD4 and virus load (VL) at month 1, 2, and 12 in the TPs and RPs.

None of the above HIV-infected people received antiretroviral therapy (ART). There was no significant difference in virus load (VL) and CD4 expression between the TPs and the RPs group in the acute infection phase (**Figure 1**). The baseline clinical characteristics of participants are presented in **Table 1**.

**Table 1:** The baseline Clinicopathological Characteristics of the Study Population.

	Gender	Ages, years	Expected days of infection, days	Viral load, copies/ml	CD4	Way of infection
The RPs-A1	male	44	52	7280	130	Homosexual transmission
The RPs-A6	male	44	223	24900	252	Homosexual transmission
The RPs-A12	male	44	386	7630	199	Homosexual transmission
The RPs-B1	male	40	31	5980	356	Homosexual transmission
The RPs-B6	male	40	144	6350	590	Homosexual transmission
The RPs-B12	male	40	312	24500	580	Homosexual transmission
The RPs-C1	male	48	30	227000	915	Homosexual transmission
The RPs-C6	male	48	192	36900	882	Homosexual transmission
The RPs-C12	male	48	315	35900	804	Homosexual transmission
The RPs-D1	male	20	30	432000	620	Homosexual transmission
The RPs-D6	male	20	198	320000	522	Homosexual transmission
The RPs-D12	male	20	368	790000	494	Homosexual transmission
The RPs-E1	male	32	40	101000	301	Homosexual transmission
The RPs-E6	male	32	117	561000	323	Homosexual transmission
The RPs-E12	male	32	341	156000	293	Homosexual transmission
The TPs-F1	male	49	54	258000	805	Homosexual transmission
The TPs-F6	male	49	157	122000	924	Homosexual transmission
The TPs-F12	male	49	340	59400	612	Homosexual transmission
The TPs-G1	male	46	43	7250	555	Homosexual transmission
The TPs-G6	male	46	178	3800	498	Homosexual transmission
The TPs-G12	male	46	346	45500	537	Homosexual transmission
The TPs-H1	male	46	30	36550	598	Homosexual transmission
The TPs-H6	male	46	153	8190	714	Homosexual transmission
The TPs-H12	male	46	348	14000	594	Homosexual transmission
The TPs-I1	male	32	37	27900	651	Homosexual transmission
The TPs-I6	male	32	140	856	518	Homosexual transmission
The TPs-I12	male	32	319	1390	369	Homosexual transmission
The TPs-J1	male	21	55	13650	548	Homosexual transmission
The TPs-J6	male	21	218	8530	565	Homosexual transmission
The TPs-J12	male	21	341	7860	507	Homosexual transmission
HC-1	male	35	0	0	–	–
HC-2	male	37	0	0	–	–
HC-3	male	28	0	0	–	–
HC-4	male	48	0	0	–	–
HC-5	male	33	0	0	–	–

RPs, rapid progressors; TPs, typical progressors; HC, health control.

**Figure 2:**
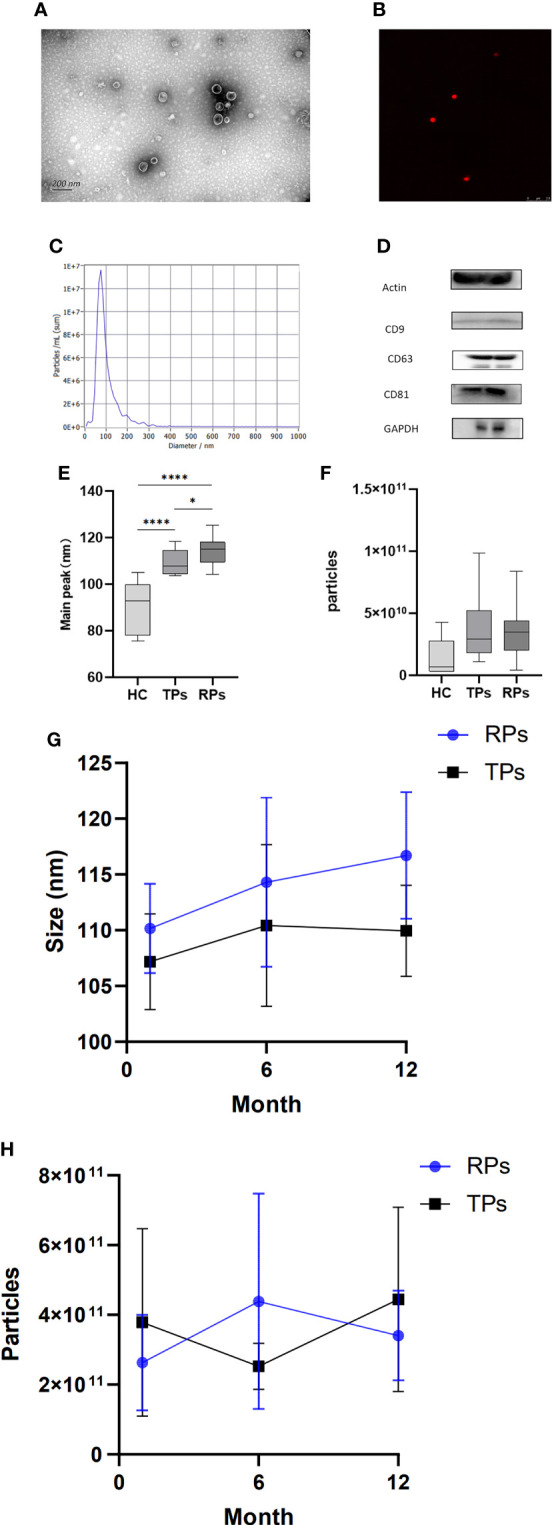
Basic characteristics and identification of exosomes in different disease progress groups. **(A)** Exosomes morphology analyzed by representative electron microscopy. Scale bar, 200 nm. **(B)** Ultrasensitive laser confocal microscopy was used to identify Exosomes (CD9-AF647). **(c)** NTA obtained the nanoparticle size distribution for plasma Exosomes. The particle size is between 30 and 150; the highest peak is observed at 70 nm. **(D)** Western blot analysis of actin, CD9, CD63, CD81, and GAPDH expression. **(E, G)** The peak size between HC, TPs, and RPs (e) and between month 1, 6, and 12 in TPs and RPs groups (g). **(F, H)** The concentration of Exosomes between HC, TPs, and RPs (f) and between month 1, 6, and 12 in TPs and RPs groups (h). * p<0.05, ****p<0.0001.

The isolated exosomes were extracted from plasma samples and analyzed by TEM, NTA, WB, and CLSM. TEM indicated that particles had bilayer membranes (**Figure 2A**); CLSM further indicated a positive expression of EV markers CD9-AF647 (**Figure 2B**). Also, over 80% of the particles had a size of 30-150nm; the peak size was 70nm (**Figure 2C**).

Next, we compared the peak size between HC, TPs, and RPs. There was a significant difference between HC and patients with acute HIV infection (**Figure 2E**). Furthermore, the peak size gradually increased in a time-dependent manner (i.e., it was lower at 3 months and higher at 12 month post-infection) in both TPs and the RPs group (**Figure 2G**). There was no significant difference in exosome concentration between the three groups; however, RPs > TPs > HC overall (**Figure 2F**). Also, there was no significant difference in exosome concentration between month 1, 2, and 12 in the TPs and the RPs groups (**Figure 2H**).

The biomarkers of exosomes, actin, CD9, CD63, CD81, and GADPH were expressed in plasma exosomes (**Figure 2D**). High-sensitivity flow cytometer and Amnis imaging flow assay were also used to identify exosomes (**Figure S1**). The small RNA sequencing of 40 samples was completed, and 1057.84 M Raw Reads were obtained, with an average of 26.45 M Raw Reads for each sample. Totally 2304 miRNAs were identified, among which 1744 were known, and 560 were newly predicted miRNAs. In addition, 18,737 miRNA target genes were predicted, and the functional annotation and enrichment analysis of the differentially expressed miRNA target genes were completed.

### The analysis of HIV-specific exosomal miRNAs expression and their function in the HC group vs. HIV-infected people

**Figure 3:**
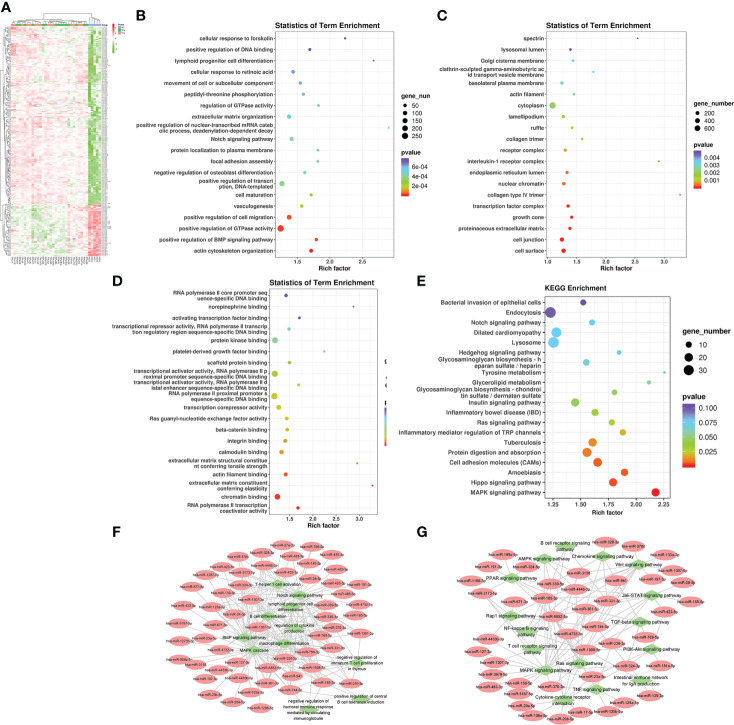
The analysis of differential HIV-specific miRNAs expression profile and their function. **(A)** The Cluster heatmap of the 208 differential exosomal miRNAs between HIV acute infected patients and HCs. **(B-D)** The GO terms interpretation for the functions of these 208 differential miRNAs. **(E)** The KEGG terms interpretation for the functions of these 208 differential miRNAs. **(F)** The miRNA target interaction regulatory network form GO terms. **(G)** The miRNA target interaction regulatory network form KEGG terms.

Firstly, we compared the HC group with HIV-infected patients. A total of 208 differentially expression miRNAs (P<0.05) were identified, among which 167 were down-regulated and 41 were up-regulated (**Figure 3A**). Next, we performed the GO and KEGG pathway enrichment analysis. According to GO enrichment analysis, positive regulation of the BMP signaling pathway, positive regulation of GTPase activity, positive regulation of cell migration, and regulation of dendrite morphogenesis were the most enriched items in the biological process (BP) (**Figure 3B**). In the cell component (CC), the significant items were cell surface, neuronal cell body, cell junction, and proteinaceous extracellular matrix (**Figure 3C**). In the molecular function (MF), the significant items were activation and repression transcription, RNA polymerase II, Ras guanyl-nucleotide exchange factor activity, and protein kinase (**Figure 3D**).

Based on KEGG analysis, glutamatergic synapse, MAPK signaling pathway, Ras signaling pathway, Hippo signaling pathway, and Insulin signaling pathway were the most common pathways (**Figure 3E**).

Then, we performed a function and signal pathway network diagram from the results of GO and KEGG to depict the function and signal pathway of differential miRNAs. From the GO BP network diagram, the results indicated these miRNAs were mainly involved in T cell differentiation, B cell differentiation, macrophage differentiation, Notch signal pathway, BMP signal pathway, and MAPK cascade (**Figure 3F**), while the KEGG network diagram indicated that these miRNAs mainly participate in B cell receptor, T cell receptor, Rap1 signal pathway and PPAR signal pathway (**Figure 3G**).

### Multiple miRNAs were defined as HIV-development characteristic miRNAs

#### The analysis of differential miRNAs between TPs and RPs

**Figure 4:**
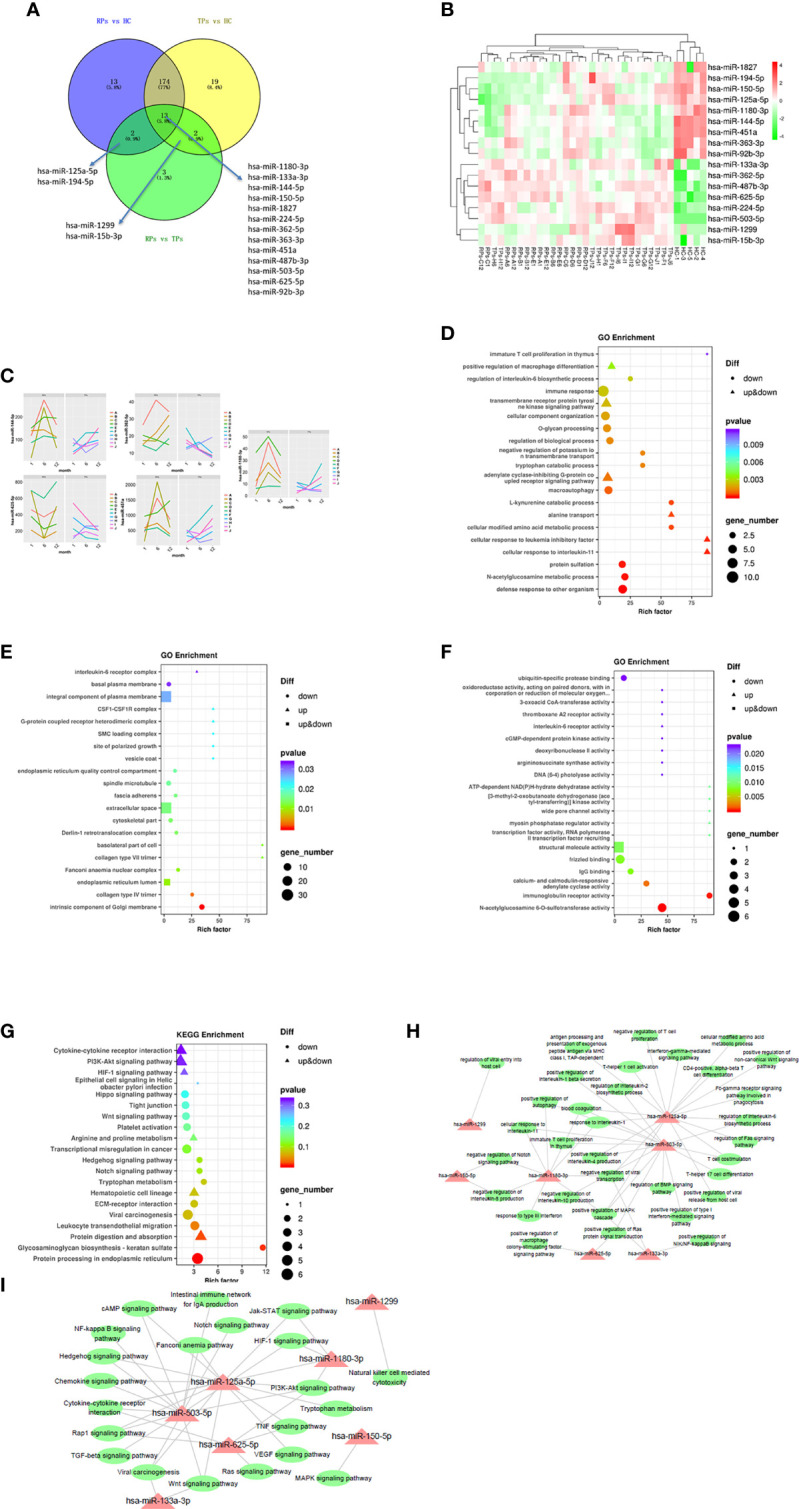
The analysis of key differential exosomal miRNAs and their variation tendency. **(A)** Venn plot showing differently expressed miRNAs between TPs and RPs at 1, 6, and 12 months. **(B)** The heatmap indicated 17 differently expressed miRNAs between RPs, TPs, and HC groups. **(C)** We depicted five miRNAs expression variations in TPs and RPs at 1, 6, and 12 months. **(D–F)** The GO terms interpretation for the functions of these 208 differential miRNAs. **(G)** The KEGG terms interpretation for the functions of these 208 differential miRNAs. **(H)** The miRNA target interaction regulatory network form GO terms. **(I)** The miRNA target interaction regulatory network form KEGG terms.

Previous research indicated that exosomal miRNAs have a significant role in the pathogenesis of HIV (28). To further investigate the specific mechanism of HIV pathogenesis in acute infection, we conducted a differential centrifugation protocol to build 35 plasma exosome groups from 15 patients and conducted an extensive analysis of the full miRNA content by small RNA sequencing. Twenty miRNAs were differentially expressed in RPs vs. the TPs. Among those, 13 miRNAs were differentially expressed in these three groups; 2 miRNAs in the RPs vs. the TPs and RPs vs. HC; 2 miRNAs in the TPs vs. RPs and TPs vs. HC. MiR-125a-5p and miR-194-5p were up-regulated in TPs and HC while down-regulated in RPs. MiR-1299 and miR-15b-3p were up-regulated in TPs while down-regulated in RPs and HC (**Figures 4A, B**). Compared with RPs, miR-1299 and miR-133a-3p were significantly down-regulated in TPs, while miR-1180-3p was significantly down-regulated in TPs (**Table 2**).

**Table 2:** Seventeen differential expression miRNAs.

#ID	P value	FDR	log2FC	regulated
hsa-miR-1299	0.000314	0.063368	2.399907	up
hsa-miR-133a-3p	0.000924	0.120669	1.275511	up
hsa-miR-194-5p	0.024285	0.518798	0.841512	up
hsa-miR-150-5p	0.020749	0.48488	0.805881	up
hsa-miR-224-5p	0.000292	0.063368	0.779132	up
hsa-miR-503-5p	0.004921	0.279297	0.732464	up
hsa-miR-125a-5p	0.003612	0.243023	0.619306	up
hsa-miR-15b-3p	0.007644	0.312881	0.588112	up
hsa-miR-1827	0.010978	0.344227	-0.59702	down
hsa-miR-92b-3p	0.001738	0.148384	-0.60224	down
hsa-miR-144-5p	0.006057	0.292292	-0.70582	down
hsa-miR-362-5p	0.001182	0.123655	-0.71238	down
hsa-miR-451a	0.018951	0.462325	-0.74516	down
hsa-miR-625-5p	0.005219	0.279297	-0.79934	down
hsa-miR-487b-3p	0.022992	0.51042	-0.80644	down
hsa-miR-363-3p	1.38E-05	0.007659	-0.86633	down
hsa-miR-1180-3p	0.001337	0.123655	-1.2494	down

A previous study indicated that the overexpression of miR-125a-5p is negatively associated with the macrophage-associated inflammatory response and may promote the M2 polarization of macrophages (29). MiR-150-5p is an immune-related miRNA that can activate T cells (30). Up-regulation of circRNA-0003528 can promote the TB-associated macrophage polarization by up-regulating the expression of CTLA4, and miR-224-5p is one of the ceRNAs in circRNA-0003528 (31). MiR-99b-3p is increased in the response of *H. capsulatum* (32). Furthermore, Wang et al found that the overexpression of miR-194-5P can enhance the production of interferon-gamma (IFN-γ) of CD8+ T cells (33). Also, Zhou et al indicated that the overexpression of miR-144-5p can reduce the viability of macrophages and inhibit the expression of TNF-alpha, IL-6, and IL-8, possibly by inhibiting the expression of TLR2 and suppressing the activation of NK-kappa B signaling (34). Another study indicated that the down-regulated miR-363-3p could promote the spread of the viral pathogen cytomegalovirus (35). Another study indicated that miR-362-5p is highly expressed in human peripheral blood NK (pNK) cells, and the overexpression of miR-362-5p can enhance the expression of IFN-γ perforin, granzyme-B, and CD107a in human primary NK cells (36).

MiR-451a in blood-circulating extracellular vesicles (Exosomes) can affect the innate immune responses of macrophages and dendritic cells (37). Specjalski and colleagues indicated that miR-625-5p could decrease cytokine secretion of Th1 cells (38). Qian et al found that overexpression of miR-625-5p can inhibit the secretion of IL-6 and TNF (39). Zhang et al found that miR-1299 can inhabit PD-L1 to enhance the immune system (40). Furthermore, Schiavinato et al indicated that miR-1299 could increase the percentage of FoxP3+ cells among CD4+CD25+/hi cells (41). These miRNAs are mainly involved in macrophage polarization, T cell activation, NK cell, IL-6, IL-8, and NK-kappa B signaling.

Next, miRNA sequencing was performed on the TPs and the RPs at 1, 6, and 12 months after virus infection (**Figure 4C**). In this study, we performed the former 17 differential miRNAs to depict images that change over time. Finally, 4 miRNAs with significant changes were selected. MiR-144-5p, miR-1180-3p, and miR-451a were gradually increased in the TPs group, while miR-362-5p and miR-625-5p were gradually down-regulated in the TPs group. In the RPs group, miR-362-5p was gradually increased. The remaining 12 miRNAs showed no obvious change trend (**Figure S2**).

#### Go and KEGG pathway enrichment analysis

Next, we performed the GO and KEGG enrichment analysis to explore the function of these miRNAs. Since strict requirements were applied for predicting the parameters of the target gene, only a few were found. GO BP term analysis indicated that RPs are characterized by a downregulated immune response, macroautophagy, defense response to other organisms, multiple amino acid metabolic changes, IL-6, and immature T cell proliferation in the thymus (**Figure 4D**). The GO CC term analysis further showed that these miRNAs are mainly involved in the IL-6 receptor complex, CSF1-CSF1R complex, and G-protein coupled heterodimeric receptor complex (**Figure 4E**). The GO MF term analysis indicated that miRNAs are mainly involved in IL-6 receptor activity, ubiquitin-specific protease binding, and cGMP-dependent protein kinase activity (**Figure 4F**). The KEGG analysis revealed that RPs are characterized by downregulation in Hippo, Wnt, Hedgehog, Notch, Tryptophan metabolism, Glycosaminoglycan biosynthesis-keratin sulfate, and protein processing in the endoplasmic reticulum (**Figure 4G**).

Consequently, we performed a miRNA target interaction regulatory network to depict the functions of miRNAs (**Figures 4H, I**). From the network diagram, in innate immunity, these miRNAs mainly involved cytokine, interferon, chemokine, and macrophage differentiation. In adaptive immunity, they mainly involved T cells. The main involved pathways included Notch, BMP, MAPK, Wnt, Hedgehog, HIF-1, PI3K-Akt, NF-kappa B, TGF-beta, Jak-STAT, Ras, and TNF signal pathways.

IL-6 is a prototypical cytokine highly correlated with inflammation and immune responses (42). A recent clinical trial indicated a higher level of IL-6 in AIDS-Kaposi’s sarcoma group compared to HIV individuals (43). In this study, IL-6 had higher expression in RPs group compared with the TPs group, which further suggested that elevated IL-6 may be an indicator of poor disease prognosis.

### Identification of the hub genes related to different HIV disease progression statuses by weighted gene co-expression network analysis (WGCNA)

**Figure 5:**
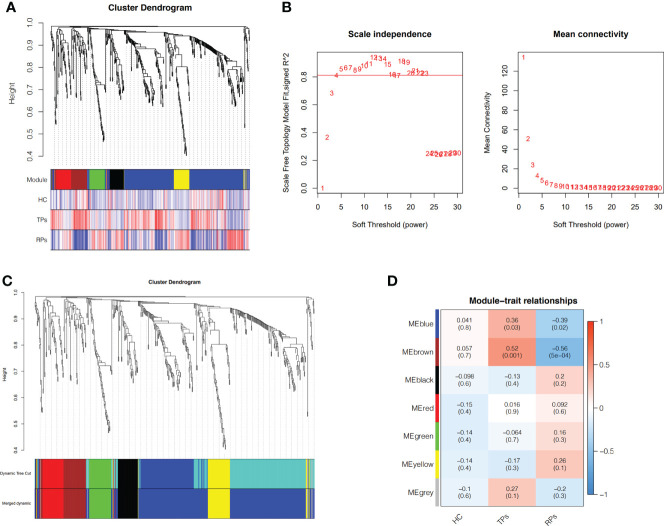
Weighted Co-Expression Network Construction and identification of key modules. **(A)** Clustering dendrogram of 35 samples with trait heatmap. **(B)** Determination of soft-threshold power in the WGCNA. **(C)** Merged module tree of miRNAs, with dissimilarity based on the topological overlap, together with assigned module colors. **(D)** Heatmap of the correlation between the module eigengenes and clinical traits of HIV. Brown and blue modules were selected for further analysis.

To identify the different progression of AIDS status, WGCNA was performed based on 35 participants. First, after deleting the outliers, the sample dendrogram and trait heatmap were built (**Figure 5A**). Second, the best soft threshold power (β) was selected as 4 to build the scaleless network (**Figure 5A**). Third, seven modules were identified by average hierarchical clustering and dynamic tree clipping (**Figure 5B**). Then, the merged module tree was constructed to express a better result (**Figure 5C**). The brown and blue modules were highly related to the progression of HIV (p<0.05); thus, these two modules were selected as clinically significant modules for further research (**Figure 5D**).

#### Enrichment analysis of GO and KEGG in the brown module

**Figure 6:**
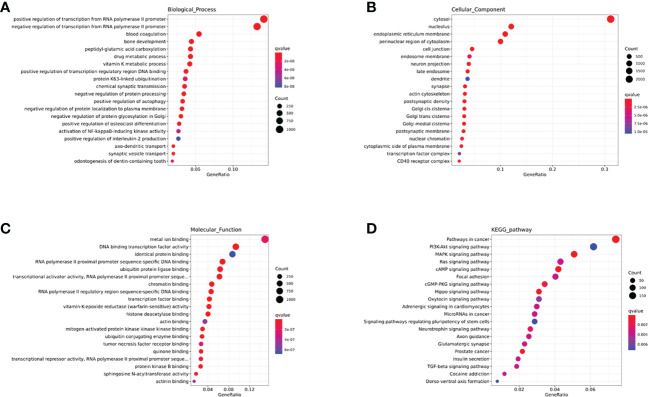
The GO and KEGG enrichment analysis interpretation for the functions of the brown module. **(A-C)** The GO terms interpretation for the functions of these 208 differential miRNAs. **(D)** The KEGG terms interpretation for the functions of these 208 differential miRNAs.

In this study, we performed an enrichment analysis of biological significance in the brown module. As seen in **Figure 6A**, the BP indicated that miRNAs were mainly associated with glycoprotein and amino acid metabolic changes, innate immunity (cytokines, interferons, chemokine, dendritic cells, and macrophages), and adaptive immunity (B cells, T helper cells, and NK cells). As seen in **Figure 6B**, the CC showed that these miRNAs were found in the cytosol, nucleolus, endoplasmic reticulum membrane, and cell conjunction. In addition, the MF suggested that these miRNAs regulate metal ion binding, DNA transcription factor activity, identical protein binding, RNA polymerase II proximal promoter sequence-specific DNA binding, and ubiquitin-protein ligase binding (in **Figure 6C**). Moreover, the KEGG indicated that these miRNAs participate in the PI3K-Akt signaling pathway, MAPK signaling pathway, RAS signaling pathway, cAMP signaling pathway, and cGMP-PKG signaling pathway (in **Figure 6D**).

#### Enrichment analysis of GO and KEGG in the blue module

**Figure 7:**
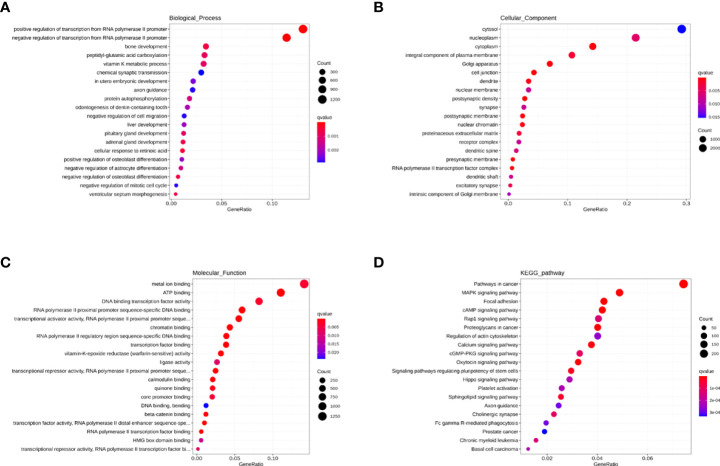
The GO and KEGG enrichment analysis interpretation for the functions for the blue module. **(A–C)** The GO terms interpretation for the functions of these 208 differential miRNAs. **(D)** The KEGG terms interpretation for the functions of these 208 differential miRNAs.

As seen in **Figure 7A**, the BP indicated that miRNAs are mainly associated with fatty acid metabolism, regulation of viral protein levels in host cells by viruses, innate immunity (cytokines, interferons, chemokine, dendritic cells, and macrophages), and adaptive immunity (B cells, T cells, and NK cells). As seen in **Figure 7B**, the CC showed that these miRNAs are found in the cytosol, nucleoplasm, cytoplasm, integral component of the plasma membrane, Golgi apparatus, and cell conjunction. In addition, the MF indicated that these miRNAs regulate metal ion binding, ATP binding, DNA binding transcription factor activity, RNA polymerase II proximal promoter sequence-specific DNA binding, and chromatin binding (**Figure 7C**). Moreover, the KEGG showed that these miRNAs participate in the MAPK signaling pathway, focal adhesion, cAMP signaling pathway, Rap1 signaling pathway, and calcium signaling pathway (**Figure 7D**).

#### The transcription factors of brown and blue module

**Figure 8:**
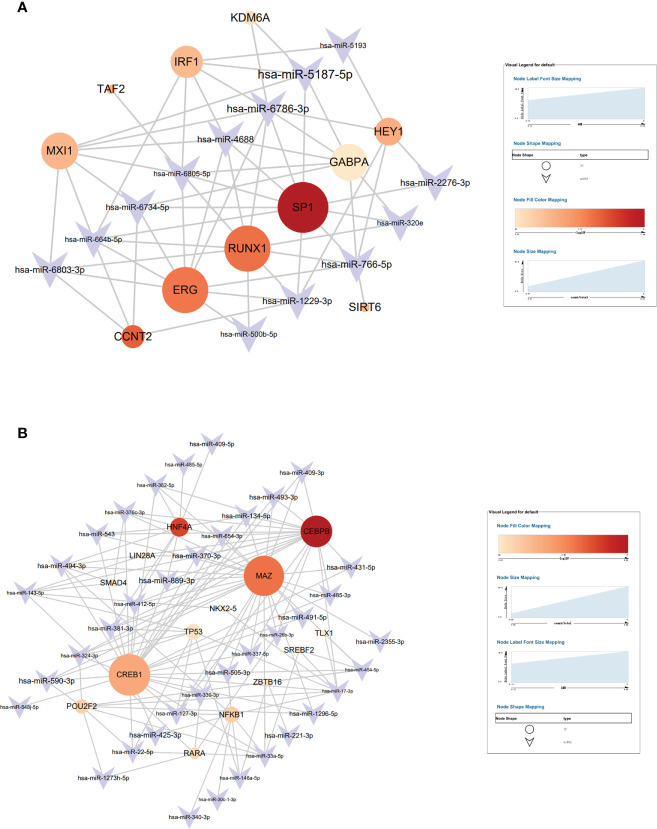
The target interaction regulatory network diagram of transcriptional factors (TF) and miRNAs of the brown module **(A)** and blue module **(B)**.

Transmit v 2.0 database was used to predict the transcription factors (TFs) of the 15 hub genes, and a network diagram of miRNAs interaction with TFs was constructed. A total of 11 TFs were selected in the brown module with the most significant TF: SP1 (**Figure 8A**) and 2 TF were selected in the blue module with the most significant TFs: CEBPB and MAZ (**Figure 8B**), and the selected criterion was P<0.05. There were 15 key miRNAs in the brown module; SP1 regulated 10 miRNAs with the most significant P-value. There were 42 key miRNAs in the blue module; CEBPB regulated 20 miRNAs, and MAZ regulated 26 miRNAs with the most significant P-value.

SP1, which is found in nearly all mammalian cell types, belongs to the strong SP/KLF family of transcription factors (44). Hotter et al indicated that IFN-inducible PYHIN protein IFI16 could reduce transcription factor Sp1 to inhabit HIV transcription (45). RUNX1 belongs to the core-binding factor family of transcription factors and is indispensable for establishing hematopoiesis in vertebrates (46). Chen et al found that lncRNA uc002yug.2 can downregulate RUNX1b and -1c to activate latent HIV-1 (47). Furthermore, ERG (ETS-Related Gene) belongs to E-twenty-six (ETS) specific factors, which is a family of more than 20 helix–loop–helix domain transcription factors (48). Studies have revealed that ERG is involved in megakaryopoiesis and T-cell development (49–52). CCAAT/enhancer-binding protein β (CEBPB) is a member of transcription factors that regulates the gene expression of immune and inflammation (53). CEBPB-deficient myeloid progenitor cells cannot produce sufficient monocytes and granulocyte-like colonies, which reduces the immune reaction (54). A previous study indicated that Myc-associated zinc finger protein (MAZ) has a significant role in immune regulation (55). Transcription factor CREB1 is related to the activation of DCs and can promote DCs to produce cytokine (56). In their study, Alantomalka et al indicated that CREB1 might promote CD4+T cells and B cells to the site of antigen presentation (57).

### The correlation between differential exosomal membrane proteins and miRNAs expression

#### Different exosomes membrane protein expressions in the 3 groups

**Figure 9:**
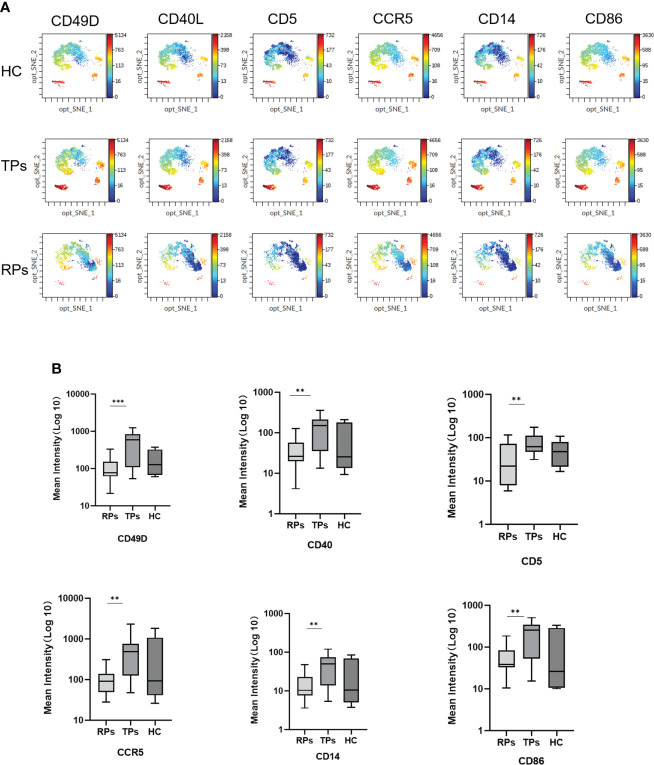
The mean intensity of six differential Exosomes membrane biomarkers. **(A)** The result of mass cytometry. **(B)** Quantification of the mean intensity. **p < 0.01, ***p < 0.001.

Mass cytometry was used to screen differential membrane proteins in the TPs and RPs. Exosomes surface proteins CD49D, CD5, CCR5, CD40, CD14, and CD86 were all higher in TPs than in RPs (**Figure 9**) (all p<0.001).

CD49D belongs to the family of integrin alpha subunits. In humans, the CD49D is associated with either CD29 or beta7 integrin and is mainly expressed in leukocytes (58). CD5 is majorly expressed in the T cell line and newborn B cells. CD5 positivity in B-cell lymphoproliferative disorders (LPD) is usually considered characteristic of chronic lymphocytic leukemia or mantle cell lymphoma (59). CCR5 is a cell membrane protein of a member of the G protein coupling factor superfamily (GPCR), expressed in multiple types of cells, and one of the main co-receptors for HIV-1 to invade the body’s cells. Besides, CCR5 can take part in the activation of T cells, so it exerts the normal function in the immune system (60).

CD14 is generally expressed in monocytes, macrophages, granulocytes, and dendritic cells (DCs). After lipopolysaccharide (LPS) or gram-negative bacteria enter the blood circulation, they bind to LPS-binding protein (LBP) to form an LPS/LBP complex. The LPS/LBP complex is then recognized and bound by mCD14 on the surface of monocytes (61). A previous study indicated that CD14 is a pattern recognition receptor (PRR) that enhances innate immune responses (61).

CD40 belongs to the costimulatory member of the tumor necrosis factor receptor (TNFR) superfamily. The interactions of B cell-expressed CD40 and its binding partner CD40L are mainly expressed in activated CD4^+^T cells, which have a significant role in promoting the germinal center formation and the production of class-switched antibodies (62). CD40 is also expressed in mature B cells, monocytes, Th cells, and dendritic cells. In Th cells, it can combine with CD40L and further co-stimulate B cell activation (62).

CD86 is expressed in professional antigen-presenting cells and can combine with CD28 to co-stimulate naïve T cells (63).

#### The correlation between the exosomes surface proteins and miRNAs

**Figure 10:**
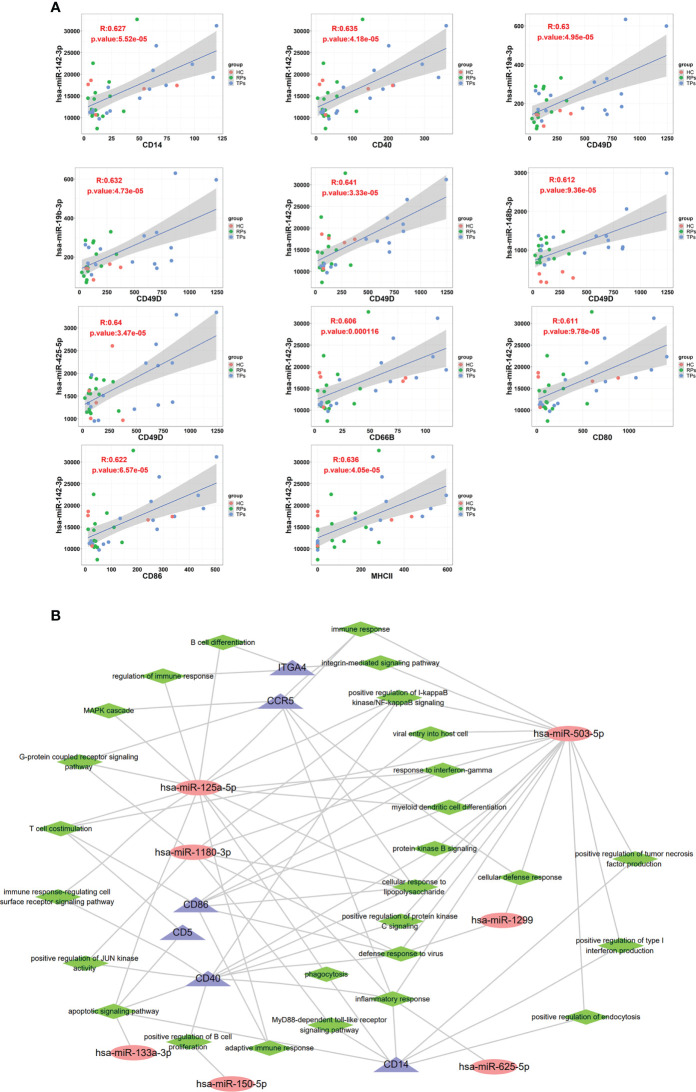
The correlation between exosome surface proteins and miRNAs. **(A)** The scatter plot of exosome surface proteins and miRNAs. **(B)** Exosomal miRNAs and surface proteins target the interaction regulatory network diagram.

In this study, we used p<0.05 and r>0.6 or r<0.6 as inclusion criteria to select the correlation between the membrane proteins and miRNAs. We filtered out low-expressed miRNAs and used high-expressed miRNAs to conduct correlation analysis with membrane proteins. Our results showed that some miRNAs are significantly positively correlated with membrane proteins, suggesting they have the same cell origin. For example, miRNA 142-3p was correlated with CD14, CD40, CD49D, CD66B, CD80, CD86, and MHC II; miR-19a-3p, miR-19b-3p, miR-148b-3p and miR-425-5p were correlated with CD49D (**Figure 10A**). CD66B is mainly expressed on neutrophils; its main functions are involved in cell adhesion and neutrophil activation (64, 65). CD80 is an essential membrane antigen for T lymphocyte activation. CD80 on the tumor is considered an essential part of antitumor CTL effector function (66). The distribution of MHC II is relatively limited, and they are mainly expressed on antigen-presenting cells such as B cells, monocytes-macrophages, and dendritic cells, and CD4+ T cells can only recognize antigen fragments bound by MHC II (67).

A previous study indicated that the overexpression of miR-142-3p can induce M2 macrophages apoptosis by transforming the growth factor-beta signaling pathway (68). Another study showed that miR-19a-3p could inhibit M1 macrophage polarization by suppressing the STAT1/IRF1 pathway (69). Lv et al. indicated exosomes miR-19b-3p could induce M1 macrophage polarization *via* NF-kappa B/SOCS-1 (70). Wang and colleagues indicated that exosomes miR-425-5p could activate PI3K/Akt signaling pathway to regulate PTEN and then induce M2 polarization of macrophages (71).

Finally, we performed an exosomes miRNA and surface protein target interaction regulatory network diagram to depict the function of miRNA and member proteins (**Figure 10B**). CD86, CD5, CD40 and CCR5 were highly correlated with miR-125a-5p, miR-1180-3p and miR-503-5p. In innate immunity, these miRNAs and member proteins are mainly involved in positive regulation of the production of type 1 interferon, interferon-γ, and inflammation response. In adaptive immunity, miRNAs are mainly involved in T cell costimulation, B cell differentiation, and positively regulating B cell proliferation. The key pathways include the integrin-mediated signaling pathway, positive regulation of NF-kappa B, MAPK cascade, G-protein coupled receptor, immune response-regulating cell surface receptor, JUN kinase, apoptotic, MyD88-dependent toll-like receptor, and positive regulation of protein kinase B and C.

## Discussion

Recently studies demonstrated that exosomes have a significant role in HIV pathology by regulating viral assembly and outflow pathways of HIV (72). Exosomes can transport viruses from infected to uninfected cells and further regulate the host’s immune response to infection (73, 74). Furthermore, exosomes can transmit disease-related products and affect the outcome of virus infections (75). Garcia-Martin et al. found that miRNAs in exosomes have tissue- or cell type-specific enrichment signatures and that exosomal member proteins can be used to trace the source of tissues or cells (76). However, so far, only a few studies have performed comprehensive exosomal miRNAs and member proteins analysis in HIV patients. Herein, we performed exosomal miRNAs sequencing and mass spectrometry to select the hub specificity exosomal miRNAs and surface protein in the progression of the RPs and the TPs, and we further explored their function.

In this study, the peak size of exosomes was gradually increased from 1 month to 12 months of HIV infection and also gradually increased from HC to TPs to RPs (**Figure 2G**), further suggesting that the peak size of exosomes may be a new marker to evaluate the progression of this disease. Moreover, by comparing the function of differential miRNAs in HC and acute HIV-infected patients, the change mainly involved cytokines, chemokines, macrophage differentiation, an intestinal immune network for IgA production, and B and T cells activity. The main pathways involved include Notch, BMP, AMPK, TGF-beta, Ras, MAPK, and TNF signal pathways. Our data suggested that decreased autophagy, amino acid metabolism, immune response, and IL-6 may be key factors associated with RPs. The function of progression in RPs and TPs related miRNA was mainly concerned with T cells, M2 macrophages, and NK cells. WGCNA was performed to select hub modules; 3 TFs (SP1, RUNX1, and ERG) were selected in the brown module and 3 TFs (CEBPB, MAZ, and CREB1) in the blue module.

Next, mass spectrometry was performed to search for the member proteins of exosomes. Next, we studied the correlation between exosome surface proteins and miRNAs. Totally six differential exosome surface proteins (CD49D, CD40, CD5, CCR5, CD14, and CD86) were identified by mass spectrometry. miRNA-142-3p was correlated with CD14, CD40, CD49D, CD66B, CD80, CD86, CD63, and MHC II; miR-19a-3p, miR-19b-3p, miR-148b-3p and miR-425-5p were correlated with CD49D. Former exosomal miRNAs were proven to be correlated with T cells and M2 macrophages, and the member proteins are mainly correlated with T cells. The infected T cells release exosomal miRNAs to affect T cells, and M2 macrophages could be the underlying mechanism. MiRNAs and proteins network show NF-kappaB signal may be the potential pathway to affect the different progression of acute HIV-infected people.

The present study has some limitations. Only bioinformatics analysis was used to examine exosomal miRNAs sequencing data and exosome surface protein. Further studies should include cell and animal experiments.

This study provides a comprehensive analysis of two different patterns of HIV progression. Our results indicated that disease-related TF has a significant role in HIV transcription alteration, metabolic alteration, innate immunity alteration, and adaptive immunity alteration. Also, disease-related miRNAs have a significant role in metabolic alteration, innate immunity, and adaptive immunity alteration, while disease-related proteins expressed in the exosome outer membrane may provide a new mechanism for clarifying the progression of acute HIV infection.

## Data availability statement

The datasets presented in this study can be found in online repositories. The name of the repository and accession number can be found below: NCBI Sequence Read Archive, PRJNA838770.

## Ethics statement

This study was approved by the Ethics Committee (seal) of Beijing Youan Hospital, Capital Medical University. Written informed consent was obtained from each subject in accordance with the Declaration of Helsinki.

## Author contributions

XL, WW and JC contributed equally to this work. WL and XL designed the experiment and wrote the manuscript. LW and CL conceived the idea and performed data analysis. JC, CC and XL conducted experiments. SL and DC revised the manuscript. All authors contributed to the article and approved the submitted version.

## Funding

This work was supported by the Natural Science Foundation of Beijing (7212172), High-level Public Health Technical Talents of Beijing(2022-2-024), National Natural Science Foundation of China(81603552), Pilot Project of Public Welfare Development and Reform of Beijing Municipal Medical Research Institutes (2019-6).

## Acknowledgments

Hongyu Liu provided helpful comments for this article.

## Conflict of interest

The authors declare that the research was conducted in the absence of any commercial or financial relationships that could be construed as a potential conflict of interest.

## Publisher’s note

All claims expressed in this article are solely those of the authors and do not necessarily represent those of their affiliated organizations, or those of the publisher, the editors and the reviewers. Any product that may be evaluated in this article, or claim that may be made by its manufacturer, is not guaranteed or endorsed by the publisher.
