# Supplementary material for: The potential role of exosomal miRNAs and membrane proteins in acute HIV-infected people
Source: Front Immunol. 2022 Aug 12;13:939504. doi: 10.3389/fimmu.2022.939504 (PMC9411714; doi:10.3389/fimmu.2022.939504)
Supplement: Supplementary file 1 [file DataSheet_1.docx]

Supplementary Material

## Supplementary Figures

##
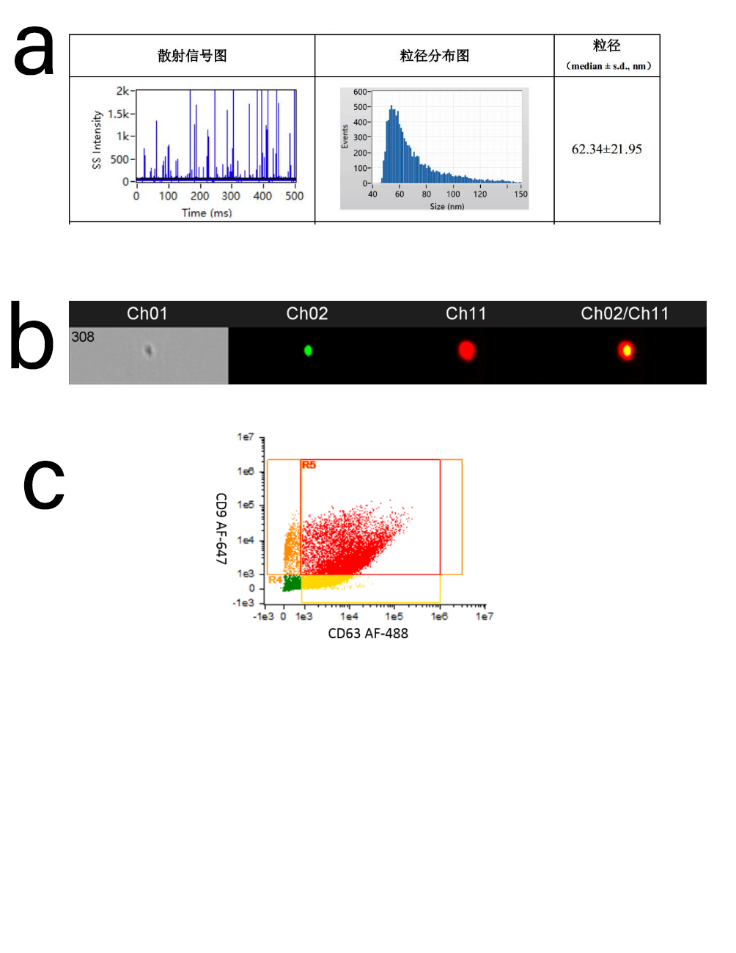


**Supplementary Figure 1.**  ***High-sensitivity flow cytometer and Amnis imaging flow assay***


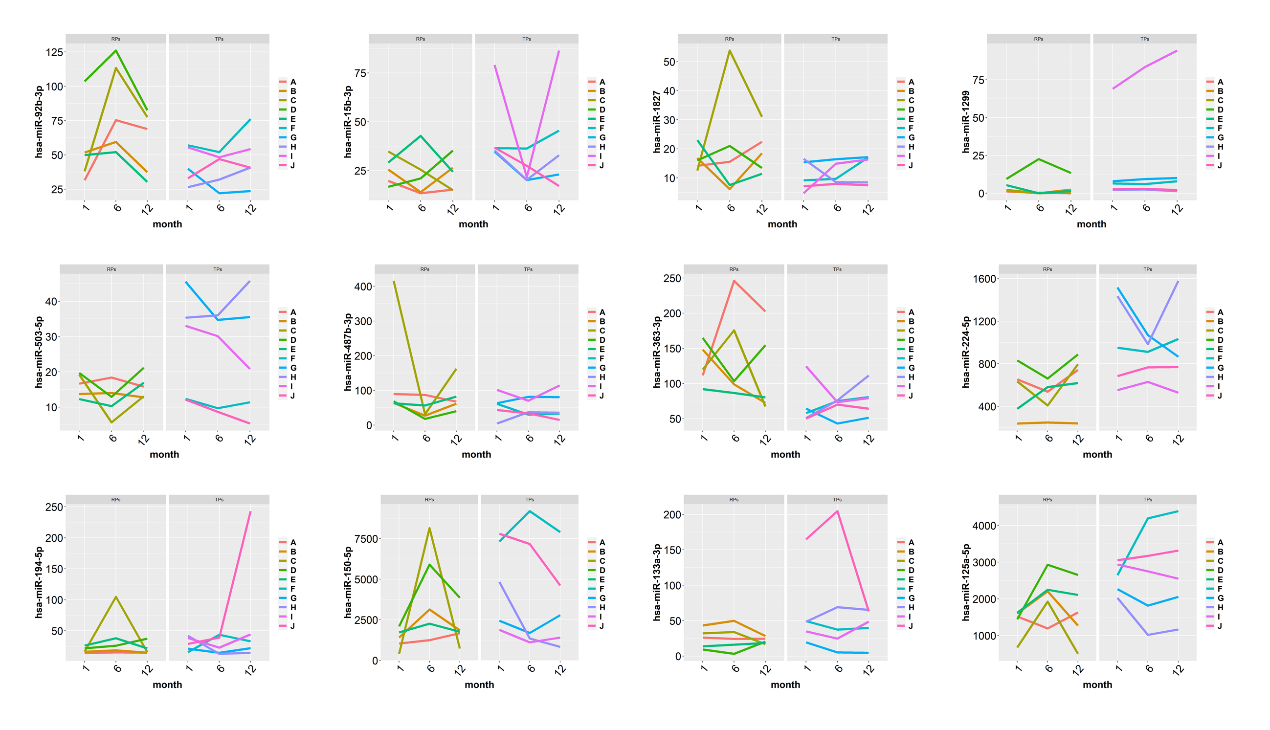


***Figure S2*** *The remain 12 miRNAs expression variation in TPs and RPs at 1, 6, and 12 month*
